# The Clinical Observation of Acupuncture Combined With Antiemetic Drugs in the Prevention and Treatment of CINV in Breast Cancer Patients

**DOI:** 10.3389/fonc.2022.888651

**Published:** 2022-07-08

**Authors:** Fanming Kong, Ziwei Wang, Na Wang, Lu Zhao, Qingyun Mei, Yongchao Yu, Dou Zhang, Xiaojiang Li, Yingjie Jia

**Affiliations:** ^1^ Department of Oncology, First Teaching Hospital of Tianjin University of Traditional Chinese Medicine, Tianjin, China; ^2^ National Clinical Research Center for Chinese Medicine Acupuncture and Moxibustion, Tianjin, China

**Keywords:** breast cancer, chemotherapy-induced nausea and vomiting, acupuncture, antiemetic drug, curative effect

## Abstract

**Objective:**

The present study aimed to explore the effectiveness of acupuncture combined with antiemetic drugs in prevention and treatment of chemotherapy-induced nausea and vomiting (CINV) among breast cancer patients receiving postoperative adjuvant chemotherapy.

**Methods:**

We retrospectively collected the clinical records of 81 postoperative breast cancer patients at our hospital from January 2021 to December 2021. These patients were divided into the acupuncture group and the control group. The efficacy of the antiemetic drugs combined with acupuncture for CINV was analysed. The primary endpoints were total, acute, and delayed nausea and vomiting grade and remission rate. Safety and overall patient quality of life were secondary endpoints.

**Results:**

During the whole observation period, compared with the control group, the frequency of nausea and vomiting was decreased in the acupuncture group (P=0.034). And the ECOG-PS score in the acupuncture group was significantly improved (P=0.004). In addition, the adverse events, such as abdominal (12.2% vs. 5.0%, P=0.252), distention (19.5% vs. 5.0%, P=0.049), and diarrhea (9.7% vs. 0, P=0.044), were decreased by acupuncture.

**Conclusions:**

Acupuncture combined with antiemetics could reduce the incidence of CINV, improve the quality of life of patients and reduce the incidence of adverse side effects of antiemetic drugs.

## Introduction

According to the annual cancer statistics report of the American Cancer Society (ACS) for 2021, the incidence rate of breast cancer was the most common cancer (1). With the development of precision medicine, although immunotherapy and targeted therapy treatment effectively prolong the survival of patients, chemotherapy still played a vital role in breast cancer treatment. According to current ASCO guidelines (2), chemotherapy was still the principal means of postoperative adjuvant, neoadjuvant and palliative treatment for breast cancer patients.

CINV, a painful side effect common to many types of chemotherapy, can lead to a series of related complications (3, 4). Without effective intervention measures, the incidence of CINV will be as high as 70% ~ 90% according to some studies (5–7). The occurrence of CINV could cause symptoms such as loss of appetite, malnutrition, and water and electrolyte disorders in patients, thus affecting the short-term efficacy and quality of life (8–10). And it could reduce patient compliance, resulting in uncompleted treatment. CINV usually included acute, delayed, explosive, refractory, and expected nausea and vomiting. Acute CINV occurs within 1-2 hours after chemotherapy and could last up to 24 hours, while the delayed CINV phase occurs more than 24 hours to 120 hours after chemotherapy (11, 12).

Nowadays, various antiemetic drugs, such as 5-hydroxytryptophan 3 (5-HT3) receptor antagonists, NK-1 receptor antagonists, phenothiazines, dopamine antagonists, antihistamines, etc, were used to prevent the incidence of CINV caused by chemotherapy. But the use of antiemetic drugs were often accompanied by some adverse reactions, which could severely influence the life quality and expectation of patients with cancers.

Hence, the novel treatment with simpling and no adverse reaction was urgently needed for CINV prevention and treatment. The acupuncture treatment was often used to treat various diseases as adjuvant therapy, It was also recommended as a supplementary therapy for the control of CINV by the National Institutes of Health (NIH) consensus statement (Citation) (13). The objective of this retrospective study was to explore the efficacy and safety of acupuncture combined with antiemetics in CINV prevention and treatment.

## Patients and methods

### Study Population

**Figure 1:**
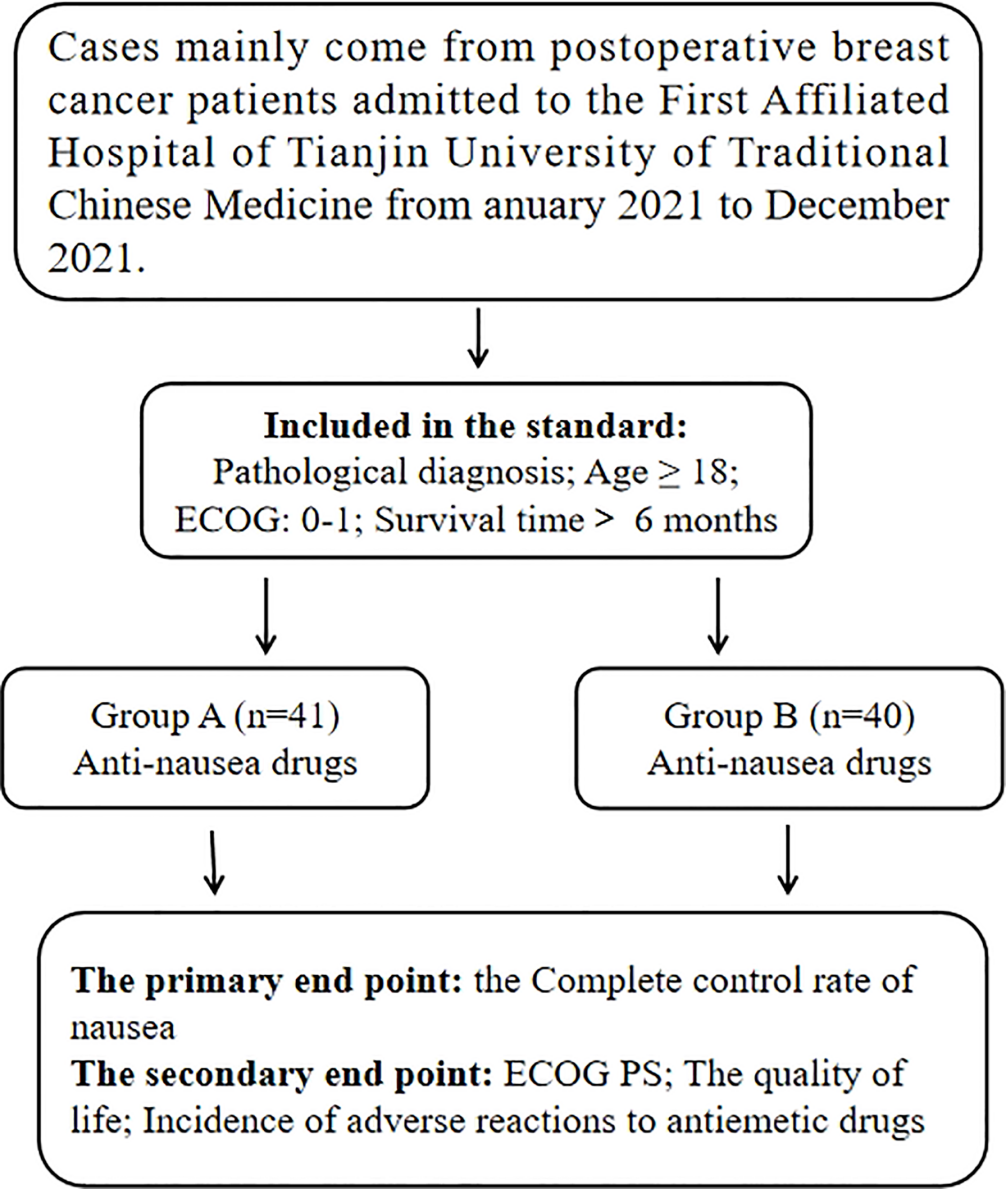
The flow chart of this study.

The 81 patients who received the AC (Cyclophosphamide and Anthracyclines) chemotherapy regimen in our hospital from January to December 2021 were enrolled. The baseline clinical data such as age, sex, ECOG-PS, cancer stage and past medical history of 81 patients were collected in **Table 1**. These patients were divided into the acupuncture group and the control group. The flow chart was showed in **Figure 1**.

**Table 1:** The basic information of 81 patients with following a curative resection for breast cancer.

	All (n=81)	Group A (n=41)	Group B (n=40)	P
Gender^*^				
Female	81	41	40	–
Age				
<50	37(45.7%)	20(48.8%)	17(42.5%)	0.570^a^
≥50	44(54.3%)	21(51.2%)	23(57.5%)	
BMI^a^				
Normal	36(44.4%)	16(39.0%)	20(50.0%)	0.320^a^
Annormal	45(55.6%)	25(61.0%)	20(50.0%)	
Stage				
I	9(11.1%)	5(12.2%)	4(10%)	0.904^b^
II	62(76.5%)	30(73.2%)	32(80%)	
III	9(11.1%)	5(12.2%)	4(10%)	
IV	1(1.2%)	1(2.4%)	0	
ECOG-PS (before)				
0	6(7.4%)	5(12.2%)	1(2.5%)	0.201^b^
1	75(92.6%)	36(87.8%)	39(97.5%)	
Alcohol				
yes	9(11.1%)	4(9.8%)	5(12.5%)	0.737^b^
no	72(88.9%)	37(90.2%)	35(87.5%)	
Motion sickness symptoms			
yes	4(4.9%)	1(2.4%)	3(7.5%)	0.359^b^
no	77(95.1%)	40(97.6%)	37(92.5%)	
Pregnancy^*^				
no	81	41	40	–

Definition of abbreviations: BMI = body mass index; ECOG-PS = Eastern Cooperative Oncology Group performance status. Data are presented as rate (%) unless otherwise indicated. P values < 0.05 are set in bold for emphasis.

a x^2^ test.

b Fisher’s exact test.

*Pregnancy and gender are constant.

The eligibility criteria for this study were as follows: (1) Age≥18 years. (2) Patients who received the AC chemotherapy regimen. (3) Eastern Cooperative Oncology Group performance status (ECOG-PS) score between 0 and 1. The exclusion criteria were as follows: (1) Patients with severe arrhythmia, severe liver damage, kidney function abnormalities, immune system, or hematopoietic system diseases. (2) Patients with intractable vomiting caused by craniocerebral disease, gastrointestinal obstruction, or other causes. (3) Before treatment, the patient happens vomiting symptoms or uses antiemetic drugs within 24 hours. (4) Incomplete original data.

### Therapeutic Regimen

The 81 patients received the AC standardly chemotherapy regimen. As everyone knows, anthracyclines is a high-risk emetic drug. Hence, it is of great significance for patients to use a standard and reasonable the antiemetic regimen to ensure the AC chemotherapy regimen’s successful completion. The guidelines suggest that the 5-HT receptor antagonist and dexamethasone combined NK1-receptor antagonist can effectively prevent and treat nausea and vomiting in patients (14–16).

Control group (A): A triple antiemetic regimen was recommended according to guidelines (17–19). The antiemetic regimen were as follows: (1) 5-HT3 receptor antagonist (palonosetron intravenously at a dose of 0.25 mg, granisetron intravenously at doses of 1 mg or orally at doses of 2 mg, or ondansetron intravenously or orally at doses of 8 mg) intravenously and orally on day 1 of chemotherapy. (2) The dexamethasone (10 mg intravenously on day 1, and 8 mg intravenously on days 2, 3, and 4 or 12 mg orally on day 1, and 8mg orally on days 2, 3, and 4) was used. (3) The NK1-receptor antagonist included intravenous fosaprepitant (150 mg on day 1) or oral aprepitant (125 mg on day 1, and 80 mg on days 2 and 3). The specific agent chosen by the primary clinician.

Acupuncture group (B): Combined acupuncture with antiemetic regimens. The specific implementation measures were as follows: (1) Acupuncture prescription: Neiguan (PC 6), Zusanli (ST 36), Zhongwan (CV 12), Gongsun G6ngsOn (SP 4), Pishu (BL 20), and Weishu (BL 21). (2) Acupuncture tools: The acupuncturists selected disposable sterile acupuncture needles (Andy brand 0. 25mm×40mm). (3) Acupuncture measures: First, acupuncture needles were inserted into the acupoints and manipulated until they achieved the “de qi” sensation. Then, leave the acupuncture needles in the body for 30 minutes. (4) Acupuncture time: From day 0 to day 5, acupuncture intervention was performed once a day. Acupuncturists are members who already hold a Chinese medicine practitioner license from the Ministry of Health of the People’s Republic of China. Registration has taken place the day before chemotherapy.

### Statistical Analysis

All analyses were performed by SPSS 25.0. The data were presented as rate (%) unless otherwise indicated. P<0.05 was considered statistically significant.

## Research Results

**Figure 2:**
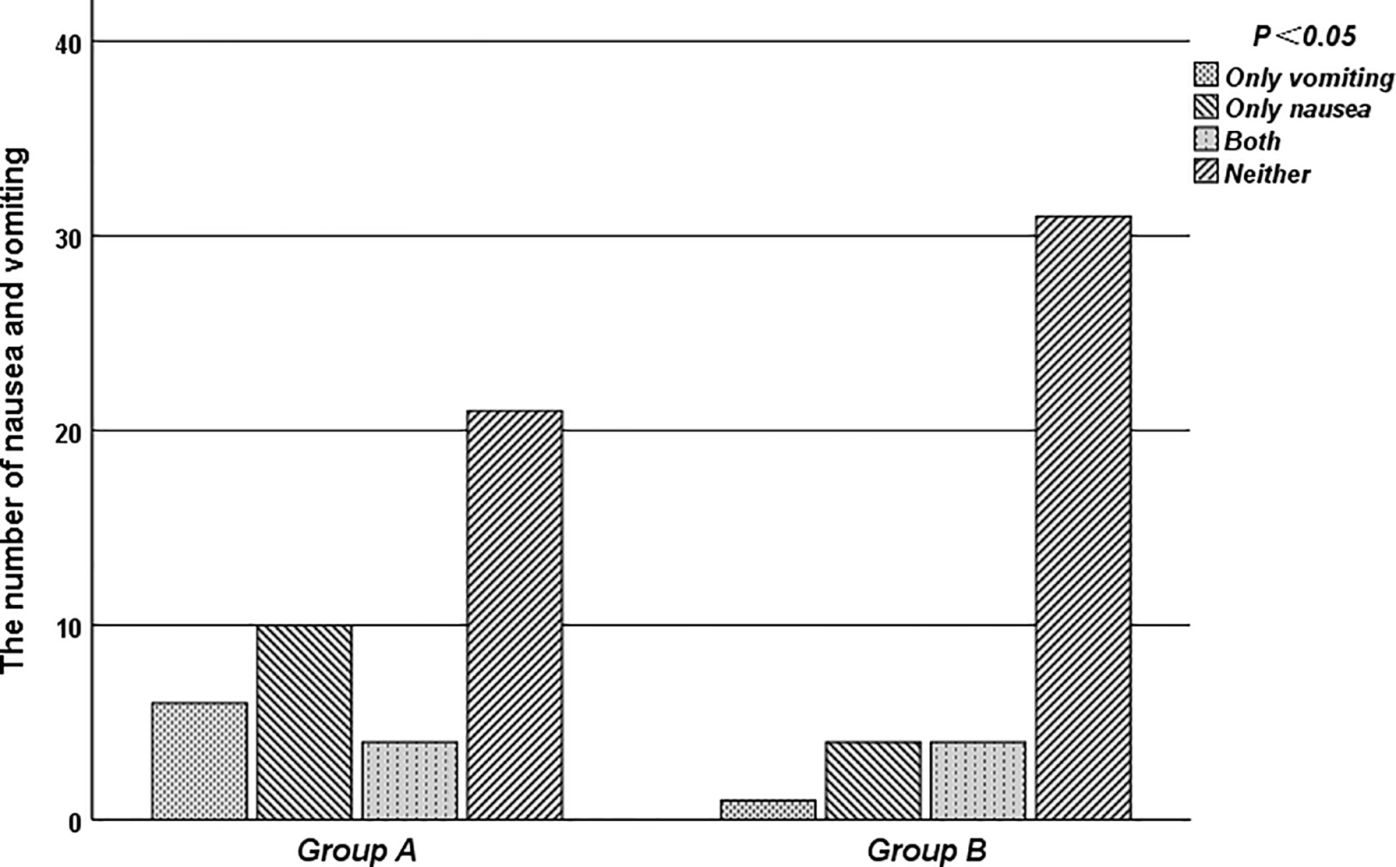
The number of nausea and vomiting in acupuncture group and control group.

As **Table 2** showed, in 0-24 hours, the frequency of vomiting was 4.9% vs. 0%, and the frequency of nausea was 7.3% vs. 5%, respectively (P=0.300). In 24-120 hours, the frequency of vomiting and nausea was 17.1% vs. 12.5%, and the frequency of nausea was 24.4% vs. 12.5%, respectively (P=0.274). In addition, compared with the control group, the frequency of nausea and vomiting was decreased in the acupuncture group during the whole observation period (P=0.034) (**Table 3**). Over the 5 survey days, the number of patients experiencing no nausea or emesis after chemotherapy or only nausea increased, and the number experiencing nausea and emesis or only emesis decreased in acupuncture group (p=0.046) (**Figure 2**). Although the patient had symptoms of nausea and vomiting, according to the CTCAE-4.03, the vomiting did not meet the classification standard, and the nausea symptoms were only graded 1 to 2, which had no significant impact on the patient’s health.

**Table 2:** The efficacy evaluation of antiemetic drugs. .

	Group A (n=41)	Group B (n=40)	*P*
**Vomiting**			
No happen	31(75.6%)	35(87.5%)	0.300^b^
0-24 h	2(4.9%)	0	
24-120 h	7(17.1%)	5(12.5%)	
0-120 h	1(2.4%)	0	
**Nausea**			
No happen	27(65.9%)	33(82.5%)	0.274^b^
0-24 h	3(7.3%)	2(5%)	
24-120 h	10(24.4%)	5(12.5%)	
0-120 h	1(2.4%)	0	
**ECOG-PS (After)**			
0	4(9.8%)	11(27.5%)	0.040^a^
1	37(90.2%)	29(72.5%)	
≥2	0	0	
**ECOG-PS** **(Intra group comparison)**	**Before/after**	**Before/after**	
0	5(12.2%)/4(9.8%)	1(2.5%)/11(27.5%)	0.157**^#c^** 0.004^*c^
1	36(87.8%)/37(90.2%)	39(97.5%)/29(72.5%)	
≥2	0	0	

Definition of abbreviations: ECOG-PS = Eastern Cooperative Oncology Group performance status. Data are presented as rate (%) unless otherwise indicated. P values < 0.05 are set in bold for emphasis.

a x^2^ test.

b Fisher’s exact test.

^C^Wilcoxon’s Sign Rank Test.

#The P values of group A.

*The P values of group B.

**Table 3:** The frequency of nausea and vomiting in the whole observation period.

	0	1	2	3	4	5	6	*P*
**Vomiting**								
Group A (n=41)	30	5	2	3	0	0	1	0.094
Group B (n=40)	35	3	1	1	0	0	0	
**Nausea**								
Group A (n=41)	26	5	2	0	4	1	3	0.034
Group B (n=40)	33	4	0	2	1	0	0	

Mann-Whitney U test is used to data analysis. P values < 0.05 are set in bold for emphasis.

The major adverse events in the two groups were headache (12.2% vs. 5%, P=0.252), diarrhea (9.7% vs. 0%, P=0.049), and constipation (19.5% vs. 5%, P=0.044) (**Table 4**). According to CTCAE-4.03, all of them were grades 1 to 2 and had no serious impact on patients. Compared with the control group, acupuncture interventions could alleviate some of the common adverse effects of antiemetic drugs. Compared with the control group, the ECOG scores of the acupuncture group were markedly improved after chemotherapy treatment (P=0.004). In addition, although the acupoint areas may occur bleeding and pain, they were usually temporary symptoms. That did not cause organic lesions nor affect the patient’s life.

**Table 4:** The grade of the adverse events.

	Group A(n=41)	Group B(n=40)	*P*
***Headache***			
Grade 0	36(87.8%)	38(95.0%)	0.252
Grade 1-2	5 (12.2%)	2 (5.0%)	
≥Grade 3	0	0	
***Constipation***			
Grade 0	33(80.5%)	38(95.0%)	0.049
Grade 1-2	8 (19.5%)	2 (5.0%)
≥Grade 3	0	0	
***Diarrhea***			
Grade 0	37(90.3%)	40(100%)	0.044
Grade 1-2	4 (9.7%)	0	
≥Grade 3	0	0	

Mann-Whitney U test is used to data analysis. P values < 0.05 are set in bold for emphasis.

## Discussion

So far, the pathogenesis of CINV was still unclear (20–23). Some studies showed that may be due to therapeutic drugs, psychological factors, or complications of advanced tumors (16). Studies also showed that the CINV was closely related to the serotonin receptors and substance P (17). The chemotherapeutic drugs stimulated the chromaffin cells in the gastrointestinal mucosa, leading to release neurotransmitters and 5-HT3. The 5-HT3 binded to its receptors, resulting in nerve impulses transmitted to the vomiting center through peripheral nerve endings to produce vomiting response. Chemotherapeutic drugs or their metabolites could directly stimulate the chemoreceptor trigger zone (CTZ), inducing neurotransmitters to produce corresponding receptors and transmit them to the vomiting center. Which can also cause the vomiting reaction (24–26).

Several prospective studies had shown that although antiemetics can improve CINV, its related adverse reactions could cause some troubles to patients. The addition of acupuncture played a vital role in reducing toxicity and enhancsing efficiency (27). The results of the present study suggested that antiemetics combined with acupuncture was more effective than antiemetics alone in the treatment of CINV. In addition, antiemetic-related adverse effects were reduced significantly. Although acupuncture may cause local skin damage during the treatment, it didn’t damage the body. At the same time, the economic burden was alleviated. So, it may be a good choice to guide the clinicians manage chemotherapy-related nausea and vomiting.

Recently, some studies also attempted to reveal the mechanism of acupuncture for CINV prevention and treatment. The related mechanism studies include (28, 29): (1) Acupuncture played an antiemetic role by affecting neurotransmitters in the CTZ. Studies showed that acupuncture could affect the transmission of endogenous opioid peptides and 5-HT, and promote the secretion of pituitary endorphins and adrenal corticosteroids in patients, which inhibited the CTZ sensing area and vomiting center, producing an antiemetic effect. (2) Chinese scholars have found that acupuncture could protect the gastrointestinal function of the rats who received chemotherapy. Acupuncture prevented the destruction and shedding of gastric mucosa and reduced the secretion of serum gastrin, declining the sensitivity of gastric mucosa to chemotherapy stimulation.

In addition, both 5-HT3 and NK-1 receptor antagonists were more effective in the CINV prevention and treatment according to some studies (20, 22). Half of the chemotherapy patients experience alonely symptoms of nausea. That because of maybe require brain awareness function to take part in the activity (24). But, after adding acupuncture to the treatment, the frequency of nausea was reduced significantly by patient feedback and scale records. So far, the mechanism of acupuncture in nausea has not been studied. That was a problem worthy of in-depth discussion.

Traditional Chinese medicine played an increasingly important role in the comprehensive treatment of cancer. In western countries, acupuncture treatment may be mainly used for pain, muscle damage, etc (30, 31). However, the number of other indications such as nausea and vomiting was still not high, which may be due to cultural differences. Traditional Chinese medicine differentiates patterns and syndromes holistically, whereas western medicine categorizes symptoms as diseases

and classifies them by their bio-chemical structures (32). In terms of Traditional Chinese medicine treatment, it is not only to eliminate symptoms, but also to strengthen the righteousness and eliminate pathogenic factors, regulate the balance yin and yang. What’s more, acupuncture treatment was not admitted to medical insurance in some western countries, which could cause an economic burden to patients (33, 34). Based on the above reasons, acupuncture treatment was different between China and the Western countries.

## Conclusions

This study indicated that acupuncture combined with antiemetics could reduce the incidence of CINV, improve the quality of life of patients and reduce the incidence of adverse side effects of antiemetic drugs. However, the mechanism of acupuncture in CINV needs to be further studied. In addition, further prospective large-sample clinical trials are needed to carry out in-depth exploration.

## Data Availability Statement

The original contributions presented in the study are included in the article/supplementary materials, further inquiries can be directed to the corresponding author/s.

## Ethics Statement

Ethical review and approval was not required for the study of human participants in accordance with the local legislation and institutional requirements.

## Author Contributions

FK and ZW contributed the central idea, analyzed most of the data, and wrote the initial draft of the paper. The remaining authors contributed to refining the ideas, carrying out additional analyses and finalizing this paper. All authors contributed to the article and approved the submitted version.

## Funding

This work is supported by the National Natural Science Foundation of China (No. 81403220), Tianjin Health and family planning-high level talent selection and training project and the National key research and development (R&D) plan (2018YFC1707400).

## Conflict of Interest

The authors declare that the research was conducted in the absence of any commercial or financial relationships that could be construed as a potential conflict of interest.

## Publisher’s Note

All claims expressed in this article are solely those of the authors and do not necessarily represent those of their affiliated organizations, or those of the publisher, the editors and the reviewers. Any product that may be evaluated in this article, or claim that may be made by its manufacturer, is not guaranteed or endorsed by the publisher.
