# Erratum: The clinical observation of acupuncture combined with antiemetic drugs in the prevention and treatment of CINV in breast cancer patients

**DOI:** 10.3389/fonc.2022.1058376

**Published:** 2022-10-13

**Authors:** Frontiers Production Office

**Affiliations:** Frontiers Media SA, Lausanne, Switzerland

**Keywords:** breast cancer, chemotherapy-induced nausea and vomiting, acupuncture, antiemetic drug, curative effect

Due to an error, the article was incorrectly published as a Review article. The correct article type is Original Research. The missing Data Availability Statement is “The original contributions presented in the study are included in the article/supplementary materials, further inquiries can be directed to the corresponding author/s.’’ and the Ethics Statement is *“*Ethical review and approval was not required for the study of human participants in accordance with the local legislation and institutional requirements*.”*

The publisher apologizes for this mistake.

The original version of this article has been updated.

